# Utilizing experimental design and desirability function in optimizing RP-HPLC method for simultaneous determination of some skeletal muscle relaxants and analgesics

**DOI:** 10.1038/s41598-024-58381-4

**Published:** 2024-05-06

**Authors:** Ayoub N. Mozayad, Marwa A. Fouad, Ehab F. Elkady

**Affiliations:** 1 Pharmaceutical Chemistry Department, Faculty of Pharmacy, Sana’a University, Sana’a, Yemen; 2 Pharmaceutical Chemistry Department, Faculty of Pharmacy, Cairo University, Kasr El-Aini Street, Cairo, 11562 Egypt; 3 Pharmaceutical Chemistry Department, School of Pharmacy, NewGiza University, Newgiza, km 22 Cairo–Alexandria Desert Road, Cairo, Egypt

**Keywords:** Experimental design, Response surface methodology, Plackett–Burman design, Box-Behnken design, Skeletal muscle relaxants, Analgesic drugs, Counterfeit, Analytical chemistry, Cheminformatics

## Abstract

An experimental design and response surface methodologies using Plackett–Burman and Box-Behnken designs were applied for selecting and optimizing the most appropriate parameters which significantly affect the separation and quantitative estimation of five skeletal muscle relaxants and four analgesic drugs (baclofen, methocarbamol, dantrolene sodium, orphenadrine citrate, cyclobenzaprine hydrochloride, ketoprofen, etoricoxib, ibuprofen, and mefenamic acid) with a relatively short duration of analysis in a single run. For the separation of the nine drugs, an INERTSIL ODS-V3-5 µm C18 column (250 × 4.6 mm I.D.) was used with the optimum mobile phase conditions (45.15 mM ammonium acetate buffer pH 5.56 adjusted with acetic acid, acetonitrile, and methanol in a ratio of 30.5:29.5:40, *v/v/v* with a flow rate of 1.5 mL/min) and UV-detection at 220 nm. The optimized method was successfully subjected to the validation steps as described in ICH guidelines for linearity, precision, accuracy, robustness, and sensitivity. The optimized and validated method was effectively applied to determine the content of the studied drugs in their pharmaceutical preparations and to expand its applicability to the counterfeit estimation of etoricoxib in different brands of tablet dosage forms.

## Introduction

The prevalence of counterfeit and substandard drugs is becoming a common worldwide problem which has been rapidly growing in recent years and affecting public health according to the WHO reports^1^. The term "counterfeit drug" refers to drugs that have been deliberately and fraudulently manufactured with mislabeling regarding their source and/or identity to become as genuine drugs such as products with the wrong or the right components, inadequate or insufficient amounts of the component(s), fraudulent packaging, or products without active ingredients^2^. The major causes of the prevalence of substandard and counterfeit drugs are the absence of government authorities’ surveillance and monitoring; lack of awareness about the dangers of fake and fraudulent drugs, especially by pharmacists and patients; the online sale of counterfeit drugs; and the economic and political situation of the country, e.g. low economic situation due to conflicts and wars^3^. The planned treatment goals and outcomes can’t be achieved if patients take counterfeit or substandard drugs. This could significantly impact their health and overall quality of life, potentially resulting in harm or even loss of life. Patients are not the only ones impacted by counterfeit and substandard pharmaceuticals; the entire health care system, medical professionals, the drug companies, drug authorities, Ministries of Health, and national economies are also affected. Common target drugs for counterfeiting include high-demand, pricey medications, e.g., chemotherapeutic agents, antivirals, antibiotics, erectile dysfunction medications, analgesics, antihistamines, weight loss aids, hormones, steroids, and antianxiety medications^4^.

Skeletal muscle relaxants have been proven to be highly effective therapeutic drugs for treating spasticity and muscle spasm, as well as being employed as surgical anesthetic adjuncts. The widespread use of skeletal muscle relaxants in medicine encouraged the creation (development) of simple, precise, accurate, sensitive, and easy-to-use methods for determining their concentration in pure prepared mixtures and pharmaceutical dosage forms^5^.

**Figure 1:**
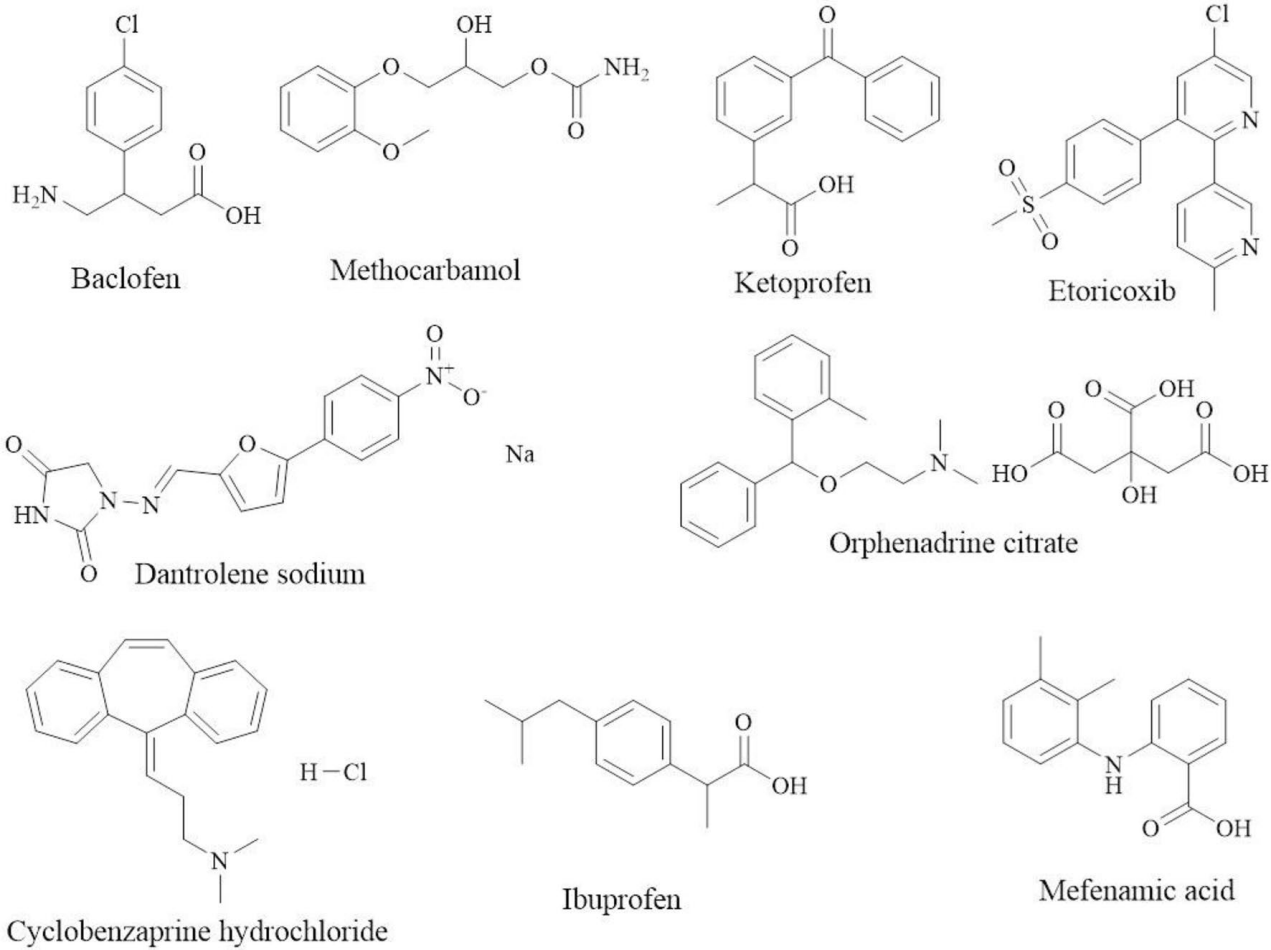
Chemical structures of the studied drugs.

Baclofen (BCL), chemically named as β-(aminomethyl)-*p*-chlorohydrocinnamic acid, is a selective agonist of the GABA-B (γ -aminobutyric acid) receptor because of its structural resemblance to the neurotransmitter γ-aminobutyric acid, which is used as a centrally acting muscle relaxant for the symptomatic treatment of a variety of neurological and musculoskeletal disorders, including muscle spasticity and multiple sclerosis^6^. Methocarbamol (MTL) is a centrally acting muscle relaxant with the chemical name ( ±)-3-(*o*-methoxyphenoxy)-1,2-propanediol-1-carbamate^7^, which is commonly used for the short-term symptomatic treatment of muscle spasms and is also used in combination with non-steroidal anti-inflammatory drugs such as ibuprofen to treat musculoskeletal disorders such as painful muscle spasms^8^. Orphenadrine citrate (ORF), chemically known as ( ±)-N,N-Dimethyl-2-[(*o*-methyl-α phenylbenzyl)oxyethylamine citrate, is a centrally acting skeletal muscle relaxant with anti-cholinergic (anti-muscarinic) and anti-histaminic effects which is used to alleviate painful muscle spasm^9^. Dantrolene sodium (DAN), chemically named 1-[[5-(p-Nitrophenyl)furfurylidene]amino]hydantoin sodium salt hydrate, is a directly acting skeletal muscle relaxant which is used intravenously for treating and preventing malignant hyperthermia and related conditions, such as neuroleptic malignant syndrome, and also orally for controlling muscle spasticity^10,11^. Cyclobenzaprine hydrochloride (CYC), chemically designated as 3-(5H-dibenzo[a,d]cyclohepten-5-ylidene)-N,N-dimethyl-1-propanamine hydrochloride, is an efficient skeletal muscle relaxant with a central action. It works primarily in the brain stem to reduce somatic motor activity, which affects the alpha and gamma motor systems. Additionally, it is utilized to treat painful muscular spasms that are frequently linked to musculoskeletal disorders^12^. Ketoprofen (KPN), chemically designated as (RS)-2-(3-benzoylphenyl)propionic acid, is a derivative of propionic acid with anti-inflammatory, analgesic, and antipyretic activities and possesses a non-selective inhibitory effect toward cyclooxygenase enzymes. It is used to treat mild to moderate pain in conditions like dysmenorrhea, migraines, post-operative pain, dental pain, and musculoskeletal and joint disorders like osteoarthritis and rheumatoid arthritis^13,14^. Etoricoxib (ETX), chemically known as 5-chloro-6’-methyl-3-[4-(methylsulfonyl)phenyl]-2,3'-bipyridine, is a non-steroidal anti-inflammatory drug (NSAID) that selectively inhibits the COX-2 enzyme and represents the second generation of COX-2 inhibitors. It is currently used to treat a number of painful disorders, including osteoarthritis, acute gout, ankylosing spondylitis, rheumatoid arthritis, dysmenorrhea, low back pain, short-term treatment of moderate pain after dental surgery, and acute postoperative pain in adults. When compared to nonselective NSAIDs, etoricoxib is typically more expensive^15,16^. Ibuprofen (IPN), chemically named (RS)-2-(4-(2-methylpropyl)phenyl)propanoic acid^17^, is a derivative of propionic acid that possesses a non-selective inhibitory effect toward cyclooxygenase enzymes and exhibits anti-inflammatory, analgesic, and antipyretic activities. It is licensed for the relief of pain and inflammation in rheumatic disease (juvenile idiopathic arthritis), mild to moderate dysmenorrhea, postoperative analgesia, migraine, dental pain, soft tissue injuries, pyrexia with discomfort, post-immunization pyrexia in infants, and other musculoskeletal disorders^13^. Mefenamic acid (MFC), chemically known as N-2,3-xylylanthranilic acid, is an anthranilic acid derivative with potent anti-inflammatory, antipyretic, and analgesic activities that is used for treating symptoms associated with menstruation, such as dysmenorrhea and menorrhagia, as well as pain and inflammation associated with osteoarthritis, rheumatoid arthritis, postoperative pain, and dental pain^18,19^. All the chemical structures of the cited drugs are shown in Fig. 1.

The development of chromatographic methods is influenced by various variables, and optimizing them is crucial for developing methods that are reliable, precise, and accurate. These methods should yield desired outcomes aligned with predefined objectives and the intended application. In accordance with ICH Q14 guidelines, there will be an increased emphasis on understanding and controlling the behavior of method variables during analytical method development. Applying the analytical quality by design (AQbD) approach, based on quality risk management (QRM) and design of experiments (DoE) principles, facilitates the understanding and control of potential method variables (PMV). Integrating DoE into chromatographic method development enhances comprehension of the relationships between critical method variables (CMVs) and critical analytical attributes (CAAs). It enables systematic exploration of the analytical design space (ADS), facilitating method optimization, robustness testing, and the identification of optimal conditions for achieving high-quality analytical outcomes. Optimizing CMVs is achieved through Response Surface Modeling (RSM) based on DoE, with exploration of the ADS ensuring the attainment of desired CAAs in accordance with the specified analytical target profile (ATP) for the chromatographic method^20^.

Plackett–Burman designs (PBD) are highly effective for screening experiments, but they are less adaptable to smoothly transitioning into optimization phases compared to two-level factorial designs. Nonetheless, their efficiency in assessing main effects with a minimal number of experimental runs makes them well-suited for robustness studies, especially when exploring slight variations from method conditions^21^.

Box-Behnken designs (BBD), a specific type of response surface methodology (RSM), demonstrate efficiency by requiring fewer experimental runs compared to designs like the central composite design with the same number of factors. This cost-effectiveness enables the effective estimation of both first- and second-order coefficients in the model, providing comprehensive insights into the relationships between factors and the response(s). However, unlike certain other RSM designs, they do not directly incorporate runs from a factorial experiment and necessitate a minimum of three factors for proper implementation^22^.

The desirability function enables the analyst to assess how effectively a combination of experimental conditions (factor levels) meets the predefined objectives for the responses. This involves achieving the optimal value for all assessed responses simultaneously and considering the researcher's priorities during the optimization process. The individual desirability (d) assesses how the settings optimize a single response, while the composite desirability (D) assesses how the settings optimize a set of responses overall. Desirability ranges from zero to one, with 1 representing the ideal combination of response values where all are within acceptable limits and zero indicating that one or more responses fall outside acceptable limits^23^.

Many analytical methods, such as colorimetry^24^, IR-spectroscopy^25,26^, Raman- spectroscopy^27^, NMR^28^, GC–MS^29–31^, TLC^32–35^, HPTLC^36^, RP-HPLC^37–41^, and LC–MS^42–44^, have been reported in the literature for the detection and determination of pharmaceutical and herbal counterfeit items.

Several liquid chromatographic methods have been published in the literature for the determination of these drugs in combinations with other drugs^14,17,45–57^, but no method has yet been reported for the simultaneous determination of these drugs as a single combined mixture.

There have been no attempts to provide a single method for separating and determining a variety of skeletal muscle relaxants with some NSAIDs. Our goal is to develop a new, versatile, economic, simple, rapid, and robust RP-HPLC method for separation and qualitative and quantitative analysis of the nine drugs in a single process. This will be achieved by employing a DoE approach and RSM, specifically utilizing PBD for screening and BBD for optimizing and estimating the robustness of the method. Statistical regression analysis was applied to investigate how different chromatographic conditions affected the separation of these drugs. The developed method was also designed with the goal of being employed in the detection and determination of any massage creams counterfeited with skeletal muscle relaxants, as well as for the counterfeit detection of etoricoxib in different brands of tablet dosage forms sold in Yemen. It is advised to use it in the quality control check for potential counterfeits of these drugs. We advise pharmaceutical companies or drug authorities to use a single analytical method to verify the quality of various pharmaceutical products during post-market monitoring because there is a huge number of different products that are taken into account for evaluation in these situations^58^.

## Experimental

### Instrumentation

The HPLC system used was an Agilent-1100 series from Japan with a G1310A-ISO pump and a G1314A-variable wavelength detector. For data collection, analysis, and instrument control, Agilent Chemstation was used. A C18 column (INERTSIL ODS-3 V, 250 mm × 4.6, 5 µm) was used for HPLC separation. A sonicator was used (Soniclean 120 T, Barton, SA, Australia). The pH was measured and adjusted using a pH meter (Jenway-3505, Essex, UK). For sample preparation and solvent filtration, non-sterile Minisart NY syringe filters and membrane filters (0.22 m and 0.45 m, SARTORIUS STEDIM BIOTECH, Germany) were used. PURELAB Option-Q was used to obtain ultra-pure water (ELGA LABWATER, UK). Data optimization and DoE were done using the Minitab-17 (Minitab, Inc., State College, Pennsylvania, USA).

### Materials and reagents

The nine estimated drugs—BCL, MTL, KPN, ORF, DAN, ETX, CYC, IPN, and MFC—were all of pharmaceutical grade. They were generously provided and confirmed by the central laboratory of drug control (Sana’a, Yemen), EIPICO Company (Tenth of Ramadan City, Egypt), PHARAONIA PHARMACEUTICALS (Cairo, Egypt), ELNASR PHARMACEUTICAL Company AMRYA PHARMACEUTICAL (Cairo, Egypt), Company (Alexandria, Egypt), and SEDICO PHARMACEUTICALS Company (Giza, Egypt) to be of purities ≥ 98%, 99.77%, 98.99%, 99.7%, 99.95%, 98.99%, 99.13%, 99.62%, and 99.98%, respectively. Acetonitrile HPLC gradient grade and methanol HPLC gradient grade (LOBA CHEMIE, Mumbai, India), ammonium acetate (MERCK, Germany), and acetic acid (El-NASR PHARMACEUTICAL CHEMICALS Co., Egypt) were used. Different pharmaceutical products were purchased from the Egyptian and Yemeni markets and are represented in Supplementary Table S1.

### Chromatographic conditions

Chromatographic separation and method validation of the nine drugs (BCL, MTL, KPN, ORF, DAN, ETX, CYC, IPN, and MFC) were performed on an Agilent 1100 HPLC system using a C18 column (INERTSIL ODS-3 V, 250 mm × 4.6, 5 µm). The optimized mobile phase consisted of 45.15 mM acetate buffer (pH 5.56 adjusted by acetic acid), acetonitrile, and methanol in the ratio of (30.5:29.5:40, *v/v/v*), which was isocratically pumped through the column at 1.5 mL/min. The column was kept at a constant temperature of 25 °C (room temperature), and the UV-detector was monitored at 220 nm. Before injecting the analytes, the column was conditioned with the mobile phase for at least 30 min to achieve equilibrium between the column and the mobile phase and to adjust the baseline.

### Preparation of stock and working standard solutions

Stock standard solutions of BCL (500 µg/mL), MTL (5000 µg/mL), KPN (2500 µg/mL), DAN (500 µg/mL), ORF (2500 µg/mL), ETX (1000 µg/mL), CYC (1000 µg/mL), IPN (5000 µg/mL), and MFC (5000 µg/mL) were separately prepared in the mobile phase and stored in a refrigerator at 2–8 °C. A working solution of ORF (1000 µg/mL) was prepared from its stock solution for method validation.

### Preparation of stock and working samples solutions

#### Tablets dosage forms sample solutions

Ten tablets of MYLOBAC, METHOCARBEX, ARCOXIA, or MULTI-RELAX were individually weighed and finely pulverized to form a homogenous powder. An accurately weighted quantity of the powder equivalent to one tablet of each dosage form was separately transferred into a 100-mL volumetric flask. To ensure that the drugs were completely dissolved, 50 mL of mobile phase was added to each flask and sonicated for at least 15 min. After cooling, each flask was completed to the mark with the mobile phase. The final step was to filter the resulting solutions through Whatman filter papers, discard the first few milliliters, and then filter them again through 0.22 μm syringe filters*.*

#### Capsules dosage forms sample solutions

The contents of 10 capsules of each of KETOLGIN, DANTRELAX, or MEFENTAN were weighed and completely mixed. An accurate portion, equivalent to the content of one capsule, was placed in a 100-mL volumetric flask. To ensure that the drugs were completely dissolved, 50 mL of mobile phase was added to each flask and shaken well by using a sonicator for at least 15 min. After cooling, each solution was diluted and completed to volume with the mobile phase. Finally, the resulting solutions were passed through Whatman filter papers, and the first few mLs were discarded and then filtered again using 0.22-μm syringe filters.

#### Ampoules sample solutions

Ten NORFLEX ampoules were thoroughly mixed. An equivalent amount of one ampoule was accurately introduced into a 100-mL volumetric flask. The volume was adjusted to the mark with the mobile phase.

#### Massage creams sample solutions

Five massage cream samples were bought from the Yemeni and Egyptian markets. One gram from each of the five massage cream products was introduced separately into a 50-mL volumetric flask. Then, 30 mL of mobile phase was added, and the solution was mixed with a vortex mixer and sonicated for 15 min to disperse the sample thoroughly. The volume was then completed with the mobile phase to the mark and passed through a suitable filter of 0.45 µm filter paper and a 2-µm pore size using a syringe filter. The first few mLs were discarded. An aliquot of 2 ml from each solution was introduced into a set of 10 ml volumetric flasks, and it was then completed to volume with the mobile phase.

#### Tablets dosage forms sample solutions for counterfeit verification

Ten tablets of ETRICIB-90, ETROPAIN-90, ENTORIK-90, ETOHEAL-90, ARCORAR-90, or E-COX-90 were weighed individually and finely pulverized to form a homogenous powder. An accurately weighted quantity of powder equivalent to one tablet of each dosage form was transferred into a 100-mL volumetric flask. To ensure that the drugs were completely dissolved, 50 mL of mobile phase was added to each flask and sonicated for at least 15 min. After cooling, each flask was completed to the mark with the mobile phase. Finally, the resulting solutions were filtered through Whatman filter papers, and the first few milliliters were discarded and then filtered again using 0.22 μm syringe filters.

### Experimental design

#### Plackett–Burman design for screening

The PBD's objective was to determine, with the fewest possible runs, the significant effects of variables on the responses and details of these variables and responses are shown in Table 1. Initially, the main effects of four parameters were estimated using the 12 runs of PBD (Table 2), which also provided a measurement of the process's inherent variability and stability.

**Table 1:** The abbreviation and definition of the studied factors and responses.

	Symbol	Definition
Factors	A	ACN% (acetonitrile%)
	B	Flow rate
	C	Buffer pH
	D	Acetate buffer concentration
Responses	t_R-9_	Retention time of mefenamic acid
	R**-**1	Resolution between baclofen and methocarbamol peaks
	R**-**2	Resolution between methocarbamol and ketoprofen peaks
	R**-**3	Resolution between ketoprofen and dantrolene sodium peaks
	R**-**4	Resolution between dantrolene sodium and orphenadrine citrate peaks
	R**-**5	Resolution between orphenadrine citrate and etoricoxib peaks
	R**-**6	Resolution between etoricoxib and cyclobenzaprine HCL peaks
	R**-**7	Resolution between cyclobenzaprine HCL and ibuprofen peaks
	R**-**8	Resolution between ibuprofen and mefenamic acid peaks

**Table 2:** Plackett–Burman design arrangement and responses.

Run order	Factors				Responses								
	A	B	C	D	t_R-9_	R-1	R-2	R-3	R-4	R-5	R-6	R-7	R-8
1	30	1.5	5.3	50	6.945	3.96	5.75	5.05	1.19	3.12	1.02	4.96	3.15
2	30	1.5	6	10	6.920	3.99	6.88	4.13	2.67	1.78	3.12	3.10	2.87
3	20	1	5.3	50	14.844	5.42	8.16	7.19	1.00	4.24	1.11	7.96	4.78
4	20	1.5	6	10	9.5660	5.01	8.28	5.59	2.42	2.37	3.06	4.26	3.80
5	30	1	6	10	10.367	4.34	7.56	4.67	3.09	1.89	3.63	3.51	3.04
6	20	1.5	6	50	9.3150	5.05	6.96	6.46	2.37	2.25	2.75	3.34	4.34
7	30	1	5.3	10	10.750	4.42	7.96	4.49	1.98	2.99	2.36	5.67	3.09
8	30	1	6	50	9.821	4.37	5.89	6.04	3.39	1.67	3.55	1.93	3.44
9	20	1	5.3	10	14.593	5.51	9.43	5.86	1.62	3.68	2.28	7.22	4.03
10	20	1.5	5.3	10	9.725	4.94	8.46	5.18	1.38	3.34	1.86	6.31	3.74
11	20	1	6	50	5.610	5.61	7.85	7.71	2.94	2.54	3.28	4.07	4.75
12	30	1.5	5.3	50	3.950	3.95	5.70	5.01	1.21	3.12	1.04	4.95	3.15

#### Box-Behnken design for factor optimization

After applying the PBD screening to select the most significant predictors on the responses (retention time of the last eluted drug and peak resolutions), the three-level four-factor BBD (Table 3), which has 27 runs with three center points, was used to assess the main and quadratic effects of the significant factors (the percentage of acetonitrile, pH of buffer, flow rate of mobile phase, and concentration of buffer) and their interaction effects on the chosen responses for further optimization of the HPLC analysis conditions.

**Table 3:** Box-Behnken design arrangement and responses.

Run order	Factors				Responses								
	A	B	C	D	t_R-9_	R-1	R-2	R-3	R-4	R-5	R-6	R-7	R-8
1	20	1.25	5.65	50	11.173	4.672	7.305	6.898	2.197	2.946	2.271	4.978	4.646
2	25	1.25	5.3	50	9.817	4.437	6.896	6.059	1.317	3.302	1.209	6.032	3.937
3	30	1.25	5.65	50	7.952	3.903	5.577	5.475	2.577	2.138	2.490	3.008	3.293
4	20	1.25	6	30	10.719	4.903	7.323	6.786	2.789	2.276	3.690	3.199	4.359
5	25	1	5.3	30	12.333	4.660	7.596	6.415	1.886	3.509	1.888	6.174	4.113
6	20	1.5	5.65	30	9.386	4.855	7.490	6.363	2.240	2.715	2.418	4.550	4.310
7	30	1.25	5.3	30	8.240	4.038	6.345	5.148	1.927	3.013	1.647	5.055	3.250
8	25	1.5	5.65	50	7.757	4.194	6.004	5.781	2.404	2.209	2.371	3.146	3.657
9	20	1.25	5.65	10	11.696	4.931	8.424	6.112	1.959	3.086	2.540	5.509	4.501
10	25	1	6	30	11.658	4.625	6.860	6.835	3.312	1.920	3.630	2.585	4.250
11	25	1.25	5.65	30	9.761	4.428	6.841	6.371	2.343	2.665	2.512	4.087	3.971
12	25	1.25	6	10	9.584	4.563	7.558	5.755	3.579	1.950	3.610	2.187	3.933
13	25	1.25	5.65	30	9.666	4.389	6.716	6.450	2.666	2.360	2.859	3.434	4.044
14	30	1.25	6	30	7.909	4.030	5.710	5.905	3.375	1.427	3.469	1.373	3.516
15	25	1.25	6	50	8.993	4.389	6.035	6.511	3.450	1.467	3.777	1.163	3.878
16	20	1	5.65	30	14.087	5.233	8.243	7.175	2.583	2.998	2.830	5.298	4.755
17	25	1.5	5.3	30	8.207	4.275	6.720	5.597	1.576	3.082	1.613	5.038	3.754
18	25	1.5	6	30	7.770	4.334	6.196	6.002	2.857	1.784	3.089	2.247	3.798
19	20	1.25	5.3	30	11.779	5.035	8.130	6.508	1.328	3.918	1.257	7.135	4.603
20	25	1.25	5.65	30	9.631	4.633	7.040	6.272	2.510	2.610	2.613	4.053	3.993
21	25	1	5.65	50	11.674	4.398	6.775	6.477	2.723	2.458	2.752	3.763	4.097
22	30	1.25	5.65	10	8.432	4.159	6.720	4.784	2.323	2.330	2.769	3.831	3.463
23	30	1.5	5.65	30	6.671	3.876	5.718	5.173	2.577	2.043	2.718	2.831	3.190
24	25	1.25	5.3	10	10.349	4.513	7.772	5.608	1.620	3.683	1.385	6.451	4.045
25	30	1	5.65	30	10.114	4.228	6.485	5.835	2.963	2.258	2.998	3.415	3.525
26	25	1	5.65	10	12.516	4.778	8.220	5.955	2.334	2.955	2.812	5.140	4.237
27	25	1.5	5.65	10	8.265	4.329	7.177	5.420	2.181	2.496	2.543	3.989	3.845

### Procedure

#### Construction of calibration curves

For calibration curves construction, different standard mixtures in the ratio of 1:15:4:1:1.2:3.6:0.6:8:5 for BCL, MTL, KPN, DAN, ORF, ETX, CYC, IPN, and MFC, respectively, were freshly prepared by introducing accurate portions of the stock standard solutions into a set of 10-mL volumetric flasks, and the volumes were diluted to the sign with the mobile phase to obtain the following concentration ranges in µg/mL: (2.5–75), (20–560), (5–240), (2.5–75), (2.5–160), (5–200), (2.5–60), (15–360), and (5–320) for BCL, MTL, KPN, ORF, DAN, ETX, CYC, IPN, and MFC, respectively. 20 µL of each solution was injected in triplicate under the optimized conditions outlined above. Nine calibration curves were obtained by drawing the relationships between the area and the corresponding concentration of each drug in µg/mL to get the necessary regression equations for each analyte.

#### Determination of the precision

For determining the precision of the proposed method, three different aliquots of standard stock solutions of the cited drugs were transferred to three 10-mL volumetric flasks, and the volume was finished to the mark with the mobile phase to get the following concentrations (20, 300, 80, 20, 24, 72, 12, 160, and 100 µg/mL), (25, 375, 100, 25, 30, 90, 15, 200, and 125 µg/mL), and (30, 450, 120, 30, 36, 108, 18, 240, and 150 µg/mL) of BCL, MTL, KPN, DAN, ORF, ETX, CYC, IPN, and MFC, which represent 80%, 100%, and 120%, respectively. Each concentration was injected three times on the same day for intraday precision and three times on three consecutive days for inter-day precision.

#### Determination of accuracy through the analysis of laboratory-prepared mixtures

Different aliquots of standard stock solutions of the nine studied drugs were transferred to a series of 10-mL volumetric flasks, and the volume was finished to the mark with the mobile phase to get the following concentration ranges in µg/mL: (5–35), (225–520), (50–140), (10–35), (18–42), (54–126), (9–21), (100–300), and (75–175) for BCL, MTL, KPN, ORF, DAN, ETX, CYC, IPN, and MFC, respectively. The process was carried out as described under the “Construction of Calibration Curves” section. The concentrations of the nine studied drugs were estimated by applying the appropriate regression equations.

#### Analysis of the cited drugs in their pharmaceutical dosage forms

For determining the nine studied drugs in their pharmaceutical dosage forms, the stock sample solutions were diluted with the mobile phase and then injected into the column in triplicate. To calculate the amounts of the cited drugs, the regression equations for each drug were used.

#### Analysis of the massage creams for counterfeit detection

After completing the extraction process and preparing the stock and working solutions of massage creams, 20 μl of each solution was injected into the column under the same ideal conditions that were chosen for the method, and then the resulting chromatogram was compared with the chromatograms resulting from the mixture of the cited drugs.

#### Analysis of the different brands of etoricoxib for counterfeit detection

For determining the counterfeit of ETX in six different brands of tablets, the stock sample solutions were diluted with the mobile phase and then injected into the column in triplicate. To calculate the amount of ETX by the standard addition method, the regression equation for ETX was used.

#### Test of system suitability

For determination of the system suitability of the proposed method, a standard mixture solution containing 50 µg/mL of the nine studied drugs was injected six times consecutively. Several parameters were examined, including column efficiency (theoretical plates), capacity factor, resolution, tailing factor, selectivity, and percentage relative standard deviation of retention times.

## Results and discussion

### Method development using analytical quality by design (AQbD) approach

Chromatographic method development is impacted by a variety of method variables and optimizing them is essential for developing methods that are robust, accurate, and precise. These methods should produce desired outcomes in line with predefined objectives and the intended application of the method. In accordance with the ICH Q14 guidelines, there will be an increased focus on understanding and controlling the behavior of method variables during the development phase of an analytical method. The understanding and control of the potential method variable (PMV) can be achieved by applying the analytical quality by design (AQbD) approach, which is based on the principles of quality risk management (QRM) and design of experiments (DoE). The application of DoE in chromatographic method development enhances the understanding of the relationships between the critical method variables (CMVs) and the critical analytical attributes (CAAs). It facilitates a systematic exploration of the analytical design space (ADS), enabling method optimization, robustness testing, and the identification of optimal conditions for achieving high-quality analytical results^20^. The optimization of CMVs can be accomplished by employing Response Surface Modeling (RSM) based on DoE. Utilizing this approach to explore the ADS enables the achievement of desired CAAs in accordance with the specified Analytical Target Profile (ATP) for the chromatographic method.

In the initial phase of implementing AQbD for the RP-HPLC method, the focus was on precisely defining the analytical target profile (ATP) and critical analytical attributes (CAAs).

The method's analytical target profile focuses on developing and validating a versatile and robust RP-HPLC method for simultaneously estimating five skeletal muscle relaxants and four analgesic drugs. The objective is to achieve a good resolution between chromatographic peaks exceeding 1.5 with a minimum run time. Consequently, the Critical Analytical Attributes (CAAs) selected for the development of the target RP-HPLC method include specific values for resolution between the studied drugs (R-1 to R-8) and retention time of the last eluted drug (t_R-9)_.

### Experimental design for optimization of chromatographic conditions

Experimental design is sequentially used to plan the experiment runs and analyze the data in order to identify the significant variables (critical method variables) that need to be reconstructed and the factor settings that produce the best possible response(s) (critical analytical attributes). The main advantage of experimental design is that it requires less time, effort, and money because only a few experimental runs are carried out. The two steps of DoE have been applied: the screening step using BPD to determine which factors are important for explaining process variation; and the second step is the optimization step using response surface design, such as BBD to create a response surface that describes the relation between the responses (CAAs) and the variables (CMVs) and determines the optimum chromatographic conditions that provide a good resolution, symmetrical peak shape, and adequate retention time.

#### Screening variables using PBD

The primary objective of our method development is to efficiently identify the most influential factors among a set of potential method variables affecting the chromatographic separation process, i.e., to efficiently select critical factors that significantly impact the method's performance. Therefore, the PBD is well-suited for this initial screening phase because it allows us to assess the main effects of multiple factors with a relatively small number of experimental runs. This screening approach enables us to focus subsequent optimization efforts on the most influential factors, simplifying the overall method development process. In summary, the rationale for choosing the PBD lies in its effectiveness as a screening tool, allowing us to efficiently identify key factors for further investigation and optimization in the development of our chromatographic method.

In this study, the PBD was applied to examine the major impacts of four parameters (A, B, C, and D), on nine responses, which are presented in Table 1. These responses included t_R-9_ (retention time of MFC) and various resolution factors between peaks of the studied drugs.

The screening variables were set at two levels (− 1, 1) as depicted in Table 2. Twelve experimental runs, resulting from the combination of four variables, were designed and randomly conducted. Response data were collected based on the designated run order, as detailed in Table 2.

**Figure 2:**
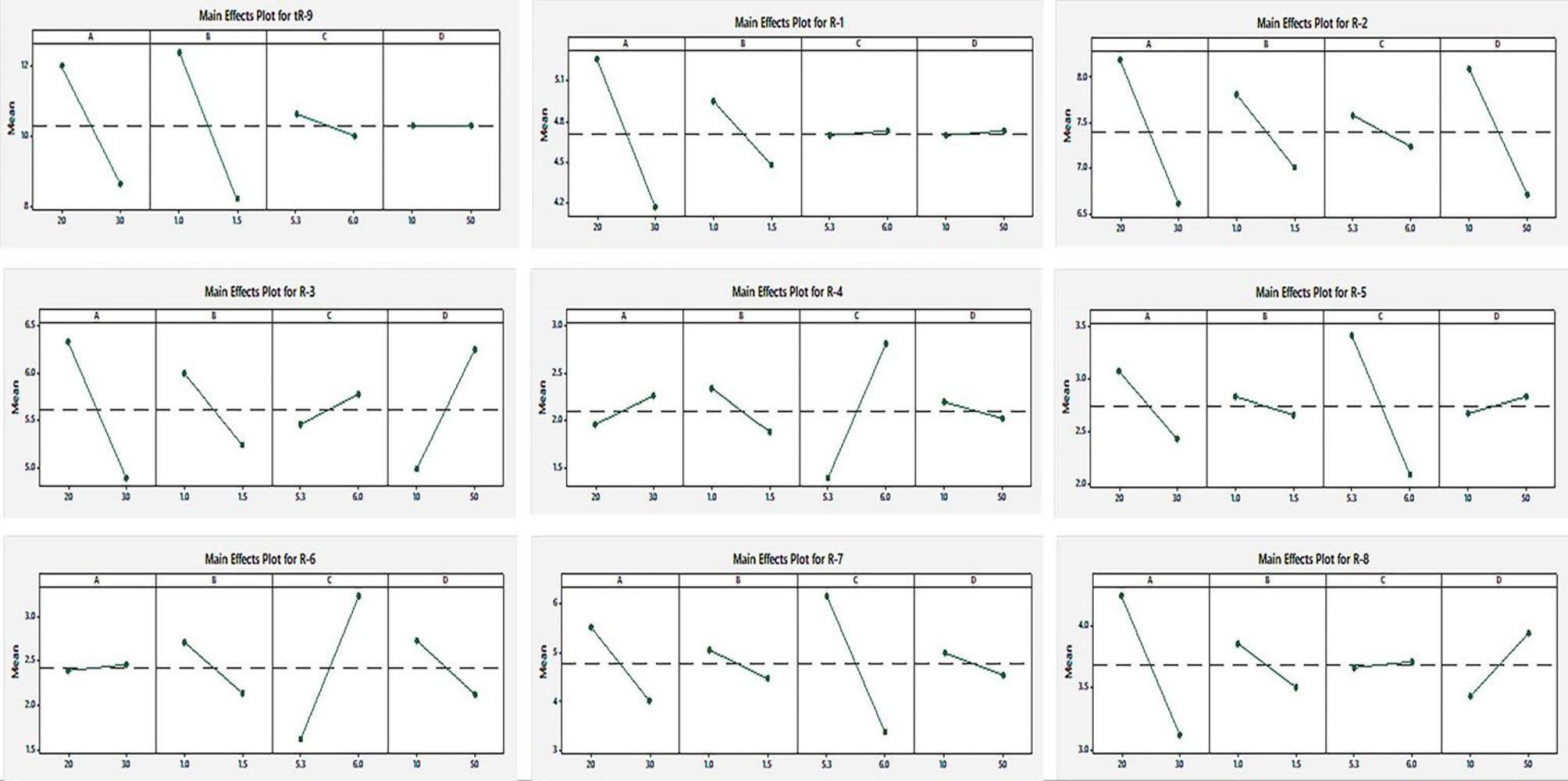
Screening of the main effects of the studied factors on the measured responses.

**Figure 3:**
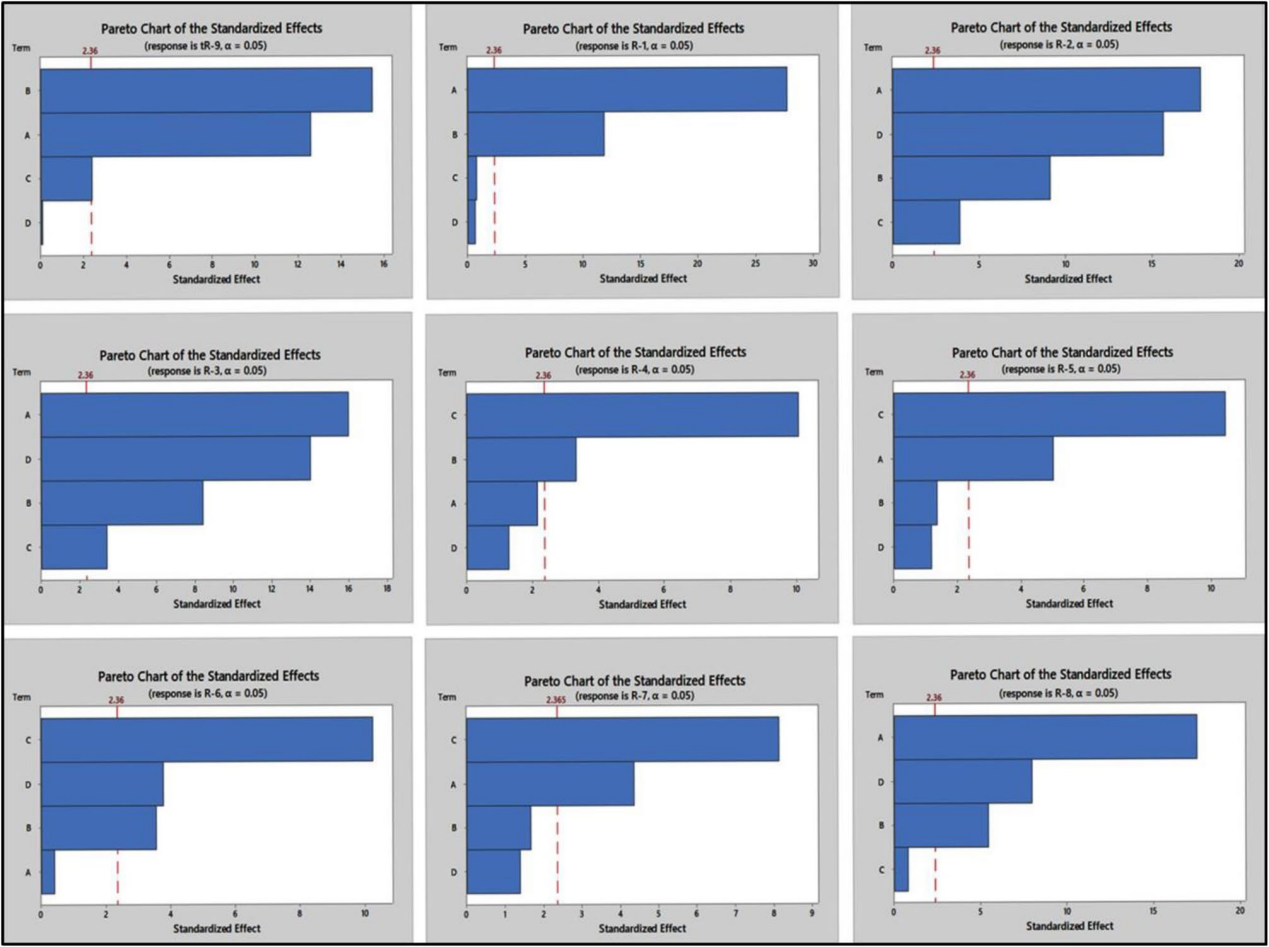
Pareto chart of the standardized effects of the studied factors on the measured responses.

The significant main effects of the variables were statistically determined through analysis of variance (ANOVA) at a 95% confidence interval, as presented in the Supplementary Table S2. A graphical representation of the main effects was done using the main effect plots shown in Fig. 2 and the Pareto chart of standardized effects. In the Pareto chart, variables that are statistically significant are those that cross the reference line, as illustrated in Fig. 3.

Based on the ANOVA results and the Pareto chart (as shown in Fig. 3), A (ACN%), B (flow rate), and C (buffer pH) all had a significantly negative effect on tR-9 (retention time). R-1 was notably affected only by A (ACN%) and B (flow rate), while R-2 and R-3 were strongly influenced by all factors. R-4 was notably affected only by B (flow rate) and C (buffer pH), while R-5 and R-7 were significantly influenced by A (ACN%) and C (buffer pH). R-6 was significantly affected by B (flow rate), C (buffer pH), and D (acetate buffer concentration), whereas R-8 was significantly affected by A (ACN%), B (flow rate), and D (acetate buffer concentration).

According to the main effect plots (Fig. 2), the Pareto chart (Fig. 3), and the ANOVA results of the PBD (Supplementary file, Table S2), it was determined that all the studied factors (A, B, C, D) were significant. Therefore, they should be optimized in the BBD to explore their main and quadratic effects, along with their interaction effects, on the desired responses^59^.

#### Optimization of factors using the BBD

BBD is a particular type of response surface methodology (RSM) that offers efficiency through a reduced number of experimental runs compared to other designs like the central composite design with the same number of factors. This makes them more cost-effective. They facilitate the effective estimation of both first- and second-order coefficients in the model, providing deeper insights into the relationships between factors and the response. However, unlike some other RSM designs, they don’t directly incorporate runs from a factorial experiment and require a minimum of three factors for proper implementation^22^.

In the response surface methodology, to extract a mathematical model depicting the relationship between factors and response variables, first-order and second-order response surface models are frequently employed. These models typically take the form of linear or quadratic polynomial functions for approximation. In simpler terms, the primary form of the first-order response surface model looks like this:

1 $${\text{y}}={\beta }_{0}+\sum_{i=n}^{n}{\beta }_{i}{x}_{i}+\varepsilon ,$$

Alternatively, the fundamental form of the second-order response surface model is expressed as follows:

2 $${\text{y}}={\beta }_{0}+\sum_{i=n}^{n}{\beta }_{i}{x}_{i}+\sum_{i=1}^{n}\sum_{j\ge i}^{n}{\beta }_{ij}{x}_{i}{x}_{j}+\varepsilon .$$

In this context, y represents the dependent variable (response); xi represents the ith component of the n-dimensional independent variable x; β_0_, βi, and βij denote the unknown parameters forming the column vector β; and ε represents the error term^60^.

The three-level, four-factor BBD with 27 runs, including three center points, was employed to optimize the experimental data and identify the ideal settings of HPLC parameters that result in good resolutions between the tested drugs with the shortest run time. The four factors selected for the optimization step were A (ACN%), B (flow rate), C (buffer pH), and D (acetate buffer concentration), and their levels are shown in Table 3. All experimental runs were carried out in a randomized order with three replications at the center points to assess the experimental error, reducing the effects of independent predictors (extraneous or uncontrollable conditions) that are not included in the study and may bias the observed results^59,61^. The inherent variance in parameters can also be estimated through randomization in order to properly draw statistical conclusions from the results of the experiment. The nine measured responses were t_R-9_, and the eight resolutions (R-1, R-2, R-3, R-4, R-5, R-6, R-7, R-8) were between the nine drugs. As a result, nine regression models were built and used to determine the surface relationship between one or more factors and the response variables. The experimental runs and measured responses are shown in Table 3, which also provides a detailed description of the BBD matrix for method optimization of four independent factors and nine responses. After collecting data from each experimental point in a chosen design, it's essential to establish a mathematical equation that describes how the response behaves with respect to the different levels of the variables studied. This means we need to estimate the parameters (β) in Eqs. (1) and (2). The regression coefficients are calculated using the least squares method, which involves minimizing the sum of squared differences between the observed values and the predicted values. Subsequently, based on these regression coefficients, a response surface models are constructed, aiding in predicting the relationship between the variables in the dataset^62^. Using regression analysis for BBD, the model regression equations that describe the model as either linear or quadratic were derived and represented in Eqs. (3–11) as follows:

3 $${t}_{R-9}= 44.1-1.229\mathrm{ A}-26.70\mathrm{ B}+3.56\mathrm{ C}-0.01449\mathrm{ D}+4.922\mathrm{ BB}-0.632\mathrm{ CC}+0.2516\mathrm{ AB}+0.1043\mathrm{ AC},$$

4 $${\text{R}}-1=7.625-0.08990\mathrm{ A}-0.6863\mathrm{ B}+0.00425\mathrm{ D }-0.00016\mathrm{ DD},$$

5 $${\text{R}}-2=15.65-0.17268\mathrm{ A}-1.625{\text{B}}-0.207{\text{C}}+0.0784{\text{D}}+0.00036\mathrm{ DD}-0.0231{\text{CD}},$$

6 $${\text{R}}-3=12.67-0.212\mathrm{ A}-1.452\mathrm{ B}-1.126\mathrm{ C}+0.06229\mathrm{ D}-0.00601\mathrm{ AA}-0.00079\mathrm{ DD}+0.0685\mathrm{ AC},$$

7 $${\text{R}}-4= -10.913 + 0.0441\mathrm{ A }- 0.655\mathrm{ B }+ 2.311\mathrm{ C},$$

8 $${\text{R}}-5= 18.558 - 0.07883\mathrm{ A }- 0.589\mathrm{ B }- 2.3054\mathrm{ C }- 0.00825\mathrm{ D},$$

9 $${\text{R}}-6= -13.021 - 0.719\mathrm{ B }+ 2.921\mathrm{ C},$$

10 $${\text{R}}-7 = 42.36 - 0.1859\mathrm{ A }- 1.525\mathrm{ B }- 5.507\mathrm{ C }-0.02090\mathrm{ D},$$

11 $${\text{R}}-8=17.59-0.5038\mathrm{ A }-0.8076\mathrm{ B}-1.815\mathrm{ C}+0.0175\mathrm{ D}+0.0729\mathrm{ AC}-0.00079\mathrm{ AD},$$

where A, B, C and D are ACN%, flow rate, buffer pH, and acetate buffer concentration, respectively. AB, AC, AD, and CD indicate the interaction between the factors, while AA, BB, CC, and DD represent the quadratic terms for each factor.

The significant linear and quadratic effects of the studied factors on various measured responses, as well as their interactive effects, are summarized in Table 4. The positive sign of a coefficient in a regression equation indicates a positive correlation between the factor and the response, while a negative sign represents a negative correlation between the factor and the response. Regression equations and ANOVA analysis revealed significant models for multiple responses. For example, according to the data in Table 4, it is evident that tR-9, R-2, and R-3 have p-values lower than 0.05 for both linear, quadratic, and interaction models (the p-value less than 0.05 indicates that the effect is significant). Additionally, R-1 shows p-values lower than 0.05 for both linear and quadratic models, indicating significance for both. Also, the linear effect of the C factor is non-significant, but its interaction effect with A (AC) is significant. Meanwhile, R-8 exhibits p-values lower than 0.05 for both linear and interaction models. However, the selected models (equations) for all responses include main effects, interaction terms, and quadratic terms. This complexity arises because when multiple significant models exist for the same response (all with p-values less than 0.05), the selected model incorporates all significant terms from each model, resulting in a more complex model. The ANOVA test was used to statistically analyze the resulting regression models. The results show that the models for the selected independent variables are significant with P-values < 0.0001, and the results of the ANOVA analysis are shown in Table 4. The p-values for all models' lack of fit suggest that the data is well-fitted by these models (a p-value of more than 0.05 is required for the models' lack of fit), and the small p-values for the linear terms, the quadratic terms, and the interactions indicate that these effects are statistically significant (for the model and the variables to be significant, the p-value must be lower than 0.05). The small p-values for the interactions (p-value < 0.05) and the squared terms (p-value < 0.05) suggest there is curvature in the response surface. The high F-values for the models and factors suggest that the models are significant at a 95% level of confidence and that the models fit the data reasonably well. Backward elimination was performed using Minitab. In this process, Minitab initially includes all variables in the model and iteratively eliminates the non-significant variable at each step. The procedure continues until all variables in the model exhibit p-values less than or equal to the specified alpha-to-remove value (0.05). Table 4 displays the R-squared, adjusted R-squared, and predicted R-squared for each model. All models exhibited R-squared values ranging from 90 to 100%, indicating that they adequately explain 90% to 100% of the variances in responses and suggesting a relatively good fit to the data. The adjusted R-squared values ranged from 89 to 100%, and the predicted R-squared values ranged from 87 to 100%. The Adjusted R-squared value serves as a more reliable measure of the model's goodness of fit than the R-squared value, especially in situations where the model incorporates an excessive number of independent variables. High values of R-squared and adjusted R-squared indicate that the data have been fitted properly by the models^39^. As noted in Table 4, the predicted R-squared values were within accepted limits with regards to the adjusted R-squared values, i.e. the difference was less than 0.2, indicating that the experimental data and the fitted model are in good agreement and demonstrating the high predictive power of models (predicted R-squared should be as near to adjusted R-squared as possible in order to make precise predictions)^59,63^.

**Table 4:** Reduced response models and statistical parameters obtained from ANOVA (after backward elimination) (AB, AC, AD, and CD indicate the interaction between the factors; AA, BB, CC, and DD represent the quadratic terms for each factor; F: F-value; P: P-value; The shaded rows represent final selected model).

Models & factors	Responses																	
	t_R-9_		R-1		R-2		R-3		R-4		R-5		R-6		R-7		R-8	
	F	P	F	P	F	P	F	P	F	P	F	P	F	P	F	P	F	P
Model	1319.59	0.000	131.50	0.000	289.90	0.000	99.78	0.000	76.90	0.000	200.15	0.000	166.92	0.000	141.14	0.000	283.01	0.000
Linear	2599.84	0.000	173.71	0.000	428.54	0.000	159.44	0.000	76.90	0.000	200.15	0.000	166.92	0.000	141.14	0.000	416.26	0.000
A	3956.18	0.000	433.55	0.000	927.67	0.000	382.52	0.000	15.36	0.001	145.40	0.000	–	–	99.59	0.000	1476.72	0.000
B	6143.88	0.000	63.16	0.000	205.26	0.000	128.33	0.000	8.47	0.008	20.32	0.000	10.00	0.004	16.75	0.000	180.11	0.000
C	173.78	0.000	–	–	123.27	0.000	40.90	0.000	206.87	0.000	609.40	0.000	323.84	0.000	428.09	0.000	0.03	0.866
D	125.50	0.000	24.42	0.000	457.95	0.000	86.02	0.000	–	–	25.47	0.000	–	–	20.14	0.000	8.18	0.010
Quadratic	45.75	0.000	4.86	0.038	14.44	0.001	28.01	0.000	–	–	–	–	–	–	–	–	–	–
AA	–	–	–	–	–	–	11.71	0.003	–	–	–	–	–	–	–	–	–	–
BB	75.47	0.000	–	–	–	–	–	–	–	–	–	–	–	–	–	–	–	–
CC	4.77	0.042	–	–	–	–	–	–	–	–	–	–	–	–	–	–	–	–
DD	–	–	4.86	0.038	14.44	0.001	51.93	0.000	–	–	–	–	–	–	–	–	–	–
Interaction	32.94	0.000	–	–	10.83	0.004	4.66	0.044	–	–	–	–	–	–	–	–	16.51	0.000
AB	49.28	0.000	–	–	–	–	–	–	–	–	–	–	–	–	–	–	–	–
AC	16.60	0.001	–	–	–	–	4.66	0.044	–	–	–	–	–	–	–	–	23.95	0.000
AD	–	–	–	–	–	–	–	–	–	–	–	–	–	–	–	–	9.08	0.007
CD	–	–	–	–	10.83	0.004	–	–	–	–	–	–	–	–	–	–	–	–
Lack-of-fit	1.87	0.403	0.26	0.963	0.29	0.946	1.61	0.451	1.50	0.476	0.43	0.874	1.23	0.544	0.75	0.715	2.00	0.386
R-sq	99.83%		95.99%		98.86%		97.35%		90.93%		97.33%		93.29%		96.25%		98.84%	
R-sq (adj)	99.75%		95.26%		98.52%		96.38%		89.75%		96.84%		92.73%		95.57%		98.49%	
R-sq (pred)	99.64%		94.01%		98.07%		94.30%		87.66%		96.05%		91.24%		94.41%		97.69%	

**Figure 4:**
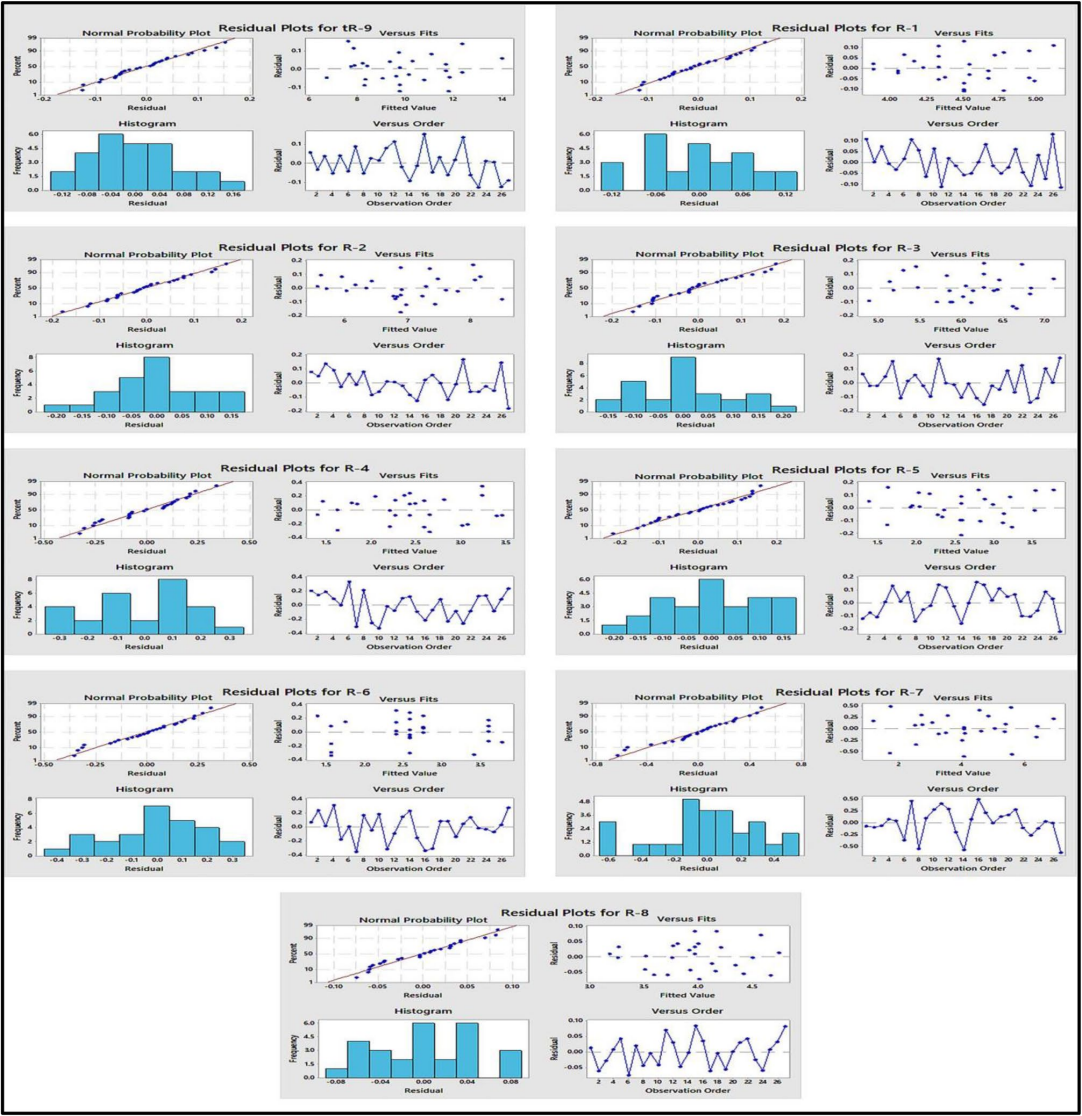
Normal probability plot, histogram, residuals versus fits and residuals versus order plots for the measured responses.

Examining residual plots was another method used to assess the model’s fit adequacy and determine whether there are any outliers, non-normality, or non-random variation^61^. The residuals are normally distributed because all points form a straight line in the normal probability plot of the residuals, as illustrated in Fig. 4, and they are randomly scattered on either side of zero, demonstrating that the errors had a constant variance and were uncorrelated with one another, as shown in residual plots versus fits and versus order in Fig. 4.

**Figure 5:**
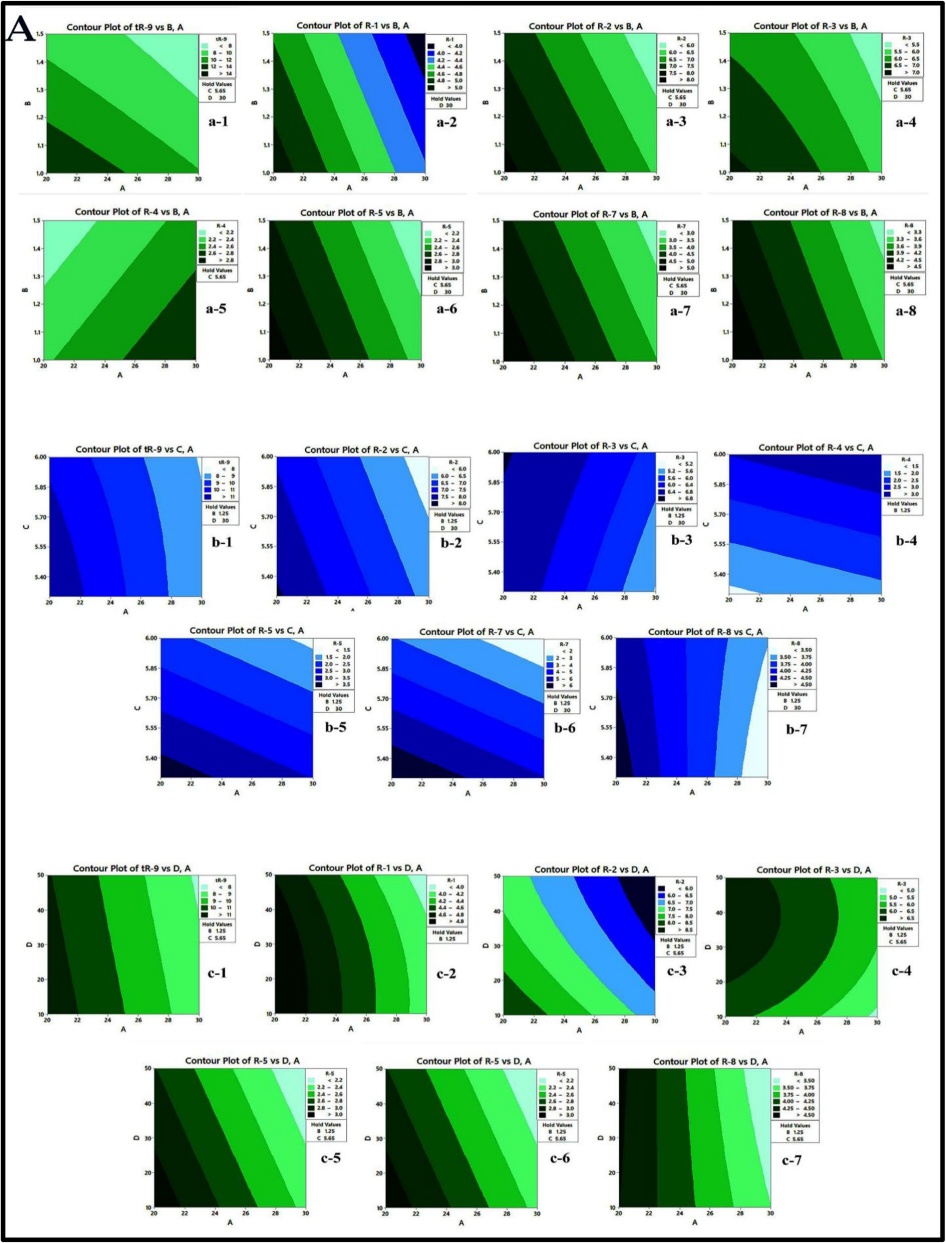

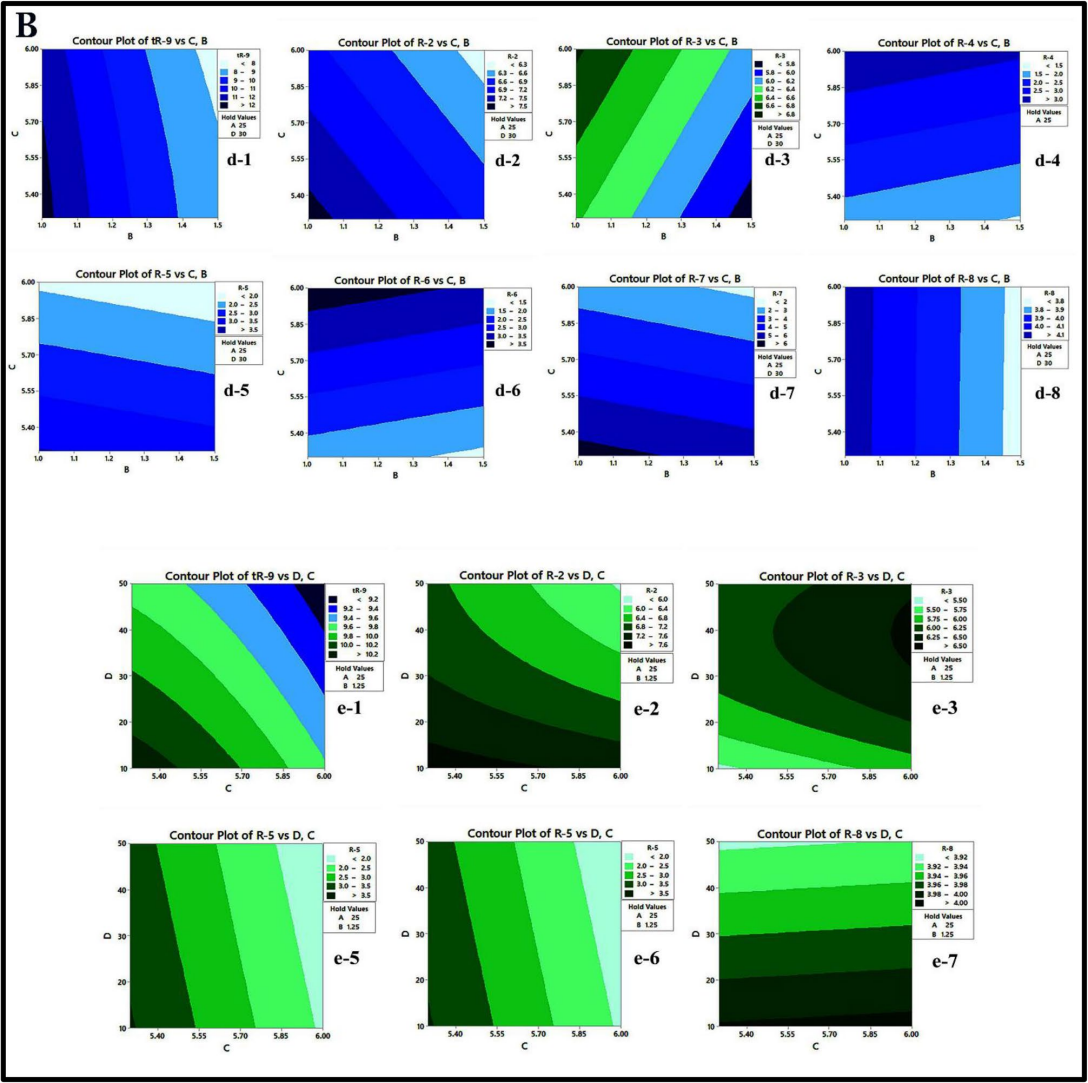
(**A**) and (**B**) Contour plots showing the interaction effects of studied factors on the measured responses.

The response surface was visually represented using 2D-contour plots (as shown in Fig. 5A and B). These plots illustrate how the response is influenced by each of the two variables and describe the shape of the response surface as shaded areas, contour lines, or both^64^ and illustrate the impact of the interaction between pairs of factors on the response while keeping the other variables at their central point values. The curvature observed in these plots indicates the non-linear effects of the factors on the response. These graphical representations facilitate the prediction of the response at any point within the analytical design space (experimental field). The combined effect of A (ACN%) and B (flow rate) on responses (tR-9, R-1, R-2, R-3, R-4, R-5, R-7, and R-8) is illustrated in Fig. 5A,a1–a8. It is observed that an increase in ACN% from 20 to 30% leads to a decrease in the previous responses, except for R4, which increases. Additionally, an increase in the flow rate from 1 to 1.5 leads to a decrease in responses. The combined effect of A (ACN%) and C (buffer pH) on responses (t_R-9_, R-2, R-3, R-4, R-5, R-7, and R-8) is illustrated in Fig. 5A,b1-b7. It is observed that an increase in buffer pH from 5.3 to 6.0 leads to a decrease in the responses t_R-9_, R-2, R-5, and R-7, while it leads to an increase in R-3, R-4, and R-8. The combined effect of A (ACN%) and D (acetate buffer concentration) on responses (t_R-9_, R-1, R-2, R-3, R-5, R-7, and R-8) is illustrated in Fig. 5A,c1-c8. In the case of the D (acetate buffer concentration), increasing the concentration from 10 to 50 mM reduces the previous responses (t_R-9_, R2, R-5, R-7, and R-8) except for R-1 and R-3. In the beginning, it leads to an increase in these two responses to a certain point, and after that, the opposite happens, i.e., an increase in the buffer concentration, it leads to a decrease in the responses R-1 and R-3.

Figure 5B,d1–d8 illustrates the combined effects of B (flow rate) and C (buffer pH) on responses (tR-9, R-2, R-3, R-4, R-5, R-6, R-7, and R-8). Additionally, the combined effects of C (buffer pH) and D (acetate buffer concentration) on responses (tR-9, R-2, R-5, R-7, and R-8) are illustrated in Fig. 5B,e1-e5.

In order to calculate the composite or combined desirability (D), the individual desirabilities for each response must first be calculated. In general, a quick analysis time with good peak resolution is recommended so that the last optimization step is based on minimizing t_R-9_, R-1, R-2, and R-8 and determining the remaining responses at target values as shown in Table 5. The following ideal conditions (the optimal solution) were optimized based on the satisfactory composite desirability that was obtained (D = 0.9998): 29.5 ACN%, flow rate of 1.5 mL/min, pH 5.56, and 45.15 mM acetate buffer, as can be seen in the optimization plot in the supplementary file, Fig. S1. For additional model validation and verification of the model’s predictive ability, an experimental run using the ideal settings of the variables (the ideal solution) was carried out, with the observed results being compared to the predicted results, as shown in Supplementary Table S3.

**Table 5:** Criteria for the optimization of the individual responses.

Response	Goal	Lower limit	Target	Upper limit	Importance
t_R-9_	Minimize	6.6713	6.6713	14.0865	1
R-1	Minimize	3.8756	3.8756	5.2325	1
R-2	Minimize	5.5767	5.5767	8.4243	1
R-3	Target	4.7842	5.1673	7.175	1
R-4	Target	1.3172	2.264	3.5789	1
R-5	Target	1.4272	2.1464	3.9183	1
R-6	Target	1.2089	2.1458	3.7767	1
R-7	Target	1.1633	2.9903	7.135	1
R-8	Minimize	3.19	3.19	4.755	1

### Application of the optimized method

#### Application to the estimation the drugs’ contents in laboratory prepared mixtures and pharmaceutical dosage forms

The method was run under ideal chromatographic conditions for quantitative determination of the nine studied drugs in their pure standard mixtures and their pharmaceutical dosage forms, and all results were in good agreement with the expected values, as shown in Table 6.

**Table 6:** Results of the regression equations and validation parameters of the optimized analysis method (R^2^: regression coefficient; *Sa* standard deviation of intercept, *Sb* standard deviation of Slope, *LOD* limit of detection, *LOQ* limit of quantitation).

Parameter		Components/data								
		BCL	MTL	KPN	DAN	ORF	ETX	CYC	IPN	MFC
Linearity range (µg/mL)		2.5–75	20–560	5–240	2.5–75	2.5–160	5–200	2.5–60	15–360	5–320
Intercept (a)		– 19.146	+ 89.085	+ 14.235	– 35.409	– 15.718	– 32.608	– 59.579	+ 106.03	– 32.33
Slope (b)		46.857	20.546	43.434	28.036	21.799	55.611	97.819	36.373	76.117
(R^2^)		0.9999	0.9998	1	0.9999	1	0.9999	0.9998	0.9999	1
(Sa)		8.186	36.246	10.681	4.576	4.559	21.484	20.107	35.984	27.698
(Sb)		0.207	0.118	0.091	0.116	0.059	0.204	0.595	0.178	0.178
LOD (µg/mL)		0.524	5.822	0.738	0.490	0.690	1.159	0.617	2.968	1.201
LOQ (µg/mL)		1.747	17.641	2.459	1.632	2.091	3.863	2.056	9.893	3.639
Intra-day (R.S.D.%)		0.17–0.87	0.07–0.85	0.14–0.87	0.34–0.75	0.32–0.99	0.16–0.64	0.24–0.61	0.10–0.58	0.18–0.57
Inter-day (R.S.D.%)		0.69–1.17	0.68–0.80	0.70–0.94	0.82–1.33	0.85–1.58	0.65–1.03	0.71–0.92	0.68–0.85	0.80–1.46
Accuracy	Lab mixture	100.21 ± 0.63	100.25 ± 0.93	100.07 ± 0.94	99.94 ± 0.53	100.52 ± 0.80	99.70 ± 0.72	100.34 ± 0.86	100.53 ± 0.65	100.92 ± 0.92
	Dosage form	97.98 ± 0.63	99.06 ± 1.14	101.97 ± 0.12	98.95 ± 0.41	101.87 ± 0.07	100.15 ± 0.82	100.75 ± 0.72	99.33 ± 1.20	99.57 ± 0.07
	Drug added	99.50 ± 0.68	100.68 ± 0.73	100.49 ± 0.70	100.75 ± 0.96	101.75 ± 0.24	100.00 ± 0.67	99.95 ± 0.38	100.41 ± 1.29	101.29 ± 0.60

#### Application to detection of counterfeits in massage creams

**Figure 6:**
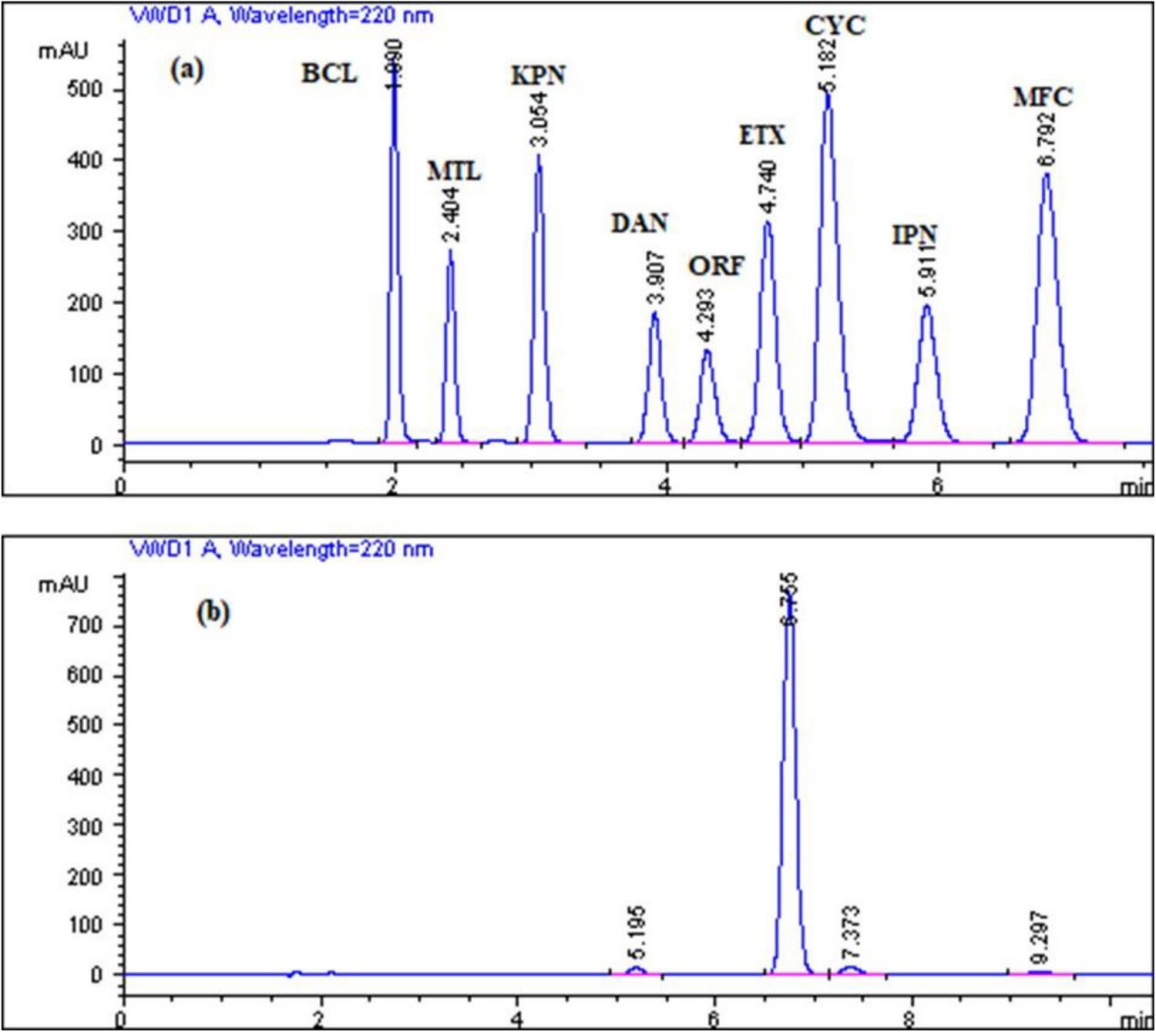
(**a**) Typical HPLC chromatogram corresponding to a laboratory prepared mixture solution of 50 µg/mL BCL (1.99 min), 50 µg/mL MET (2.404 min), 50 µg/mL KPN (3.054 min), 50 µg/mL DAN (3.907 min), 50 µg/mL ORF (4.293 min), 50 µg/mL ETX (4.740 min), 50 µg/mL CYC (5.182 min), 50 µg/mL IPN (5.911 min) and 50 µg/mL MFC (6.792 min) under the optimized chromatographic conditions; (**b**) HPLC chromatogram corresponding to ROTMOOV massage cream sample solution under the optimum assay conditions.

The optimized HPLC method was applied to detect whether the massage creams were adulterated with skeletal muscle relaxants and analgesic drugs by comparing the retention times of the peaks that were eluted from the massage creams to the retention times of the studied drug standards, as shown in Fig. 6. It was found that all massage cream products had no peaks at the retention times of the studied drug standards except ROTMOOV massage cream, which had a small peak at the retention time of CYC. When the studied ROTMOOV massage cream solution was spiked with DAN, there was no change in the height of the peak of the ROTMOOV massage cream, and this confirmed the absence of potential counterfeiting with the suspected drug in the massage creams.

#### Application to detection of counterfeits in etoricoxib tablets

The optimized method was applied to estimate the drugs' contents in 6 different brands of etoricoxib. According to the quantitative analysis of six different brands of etoricoxib coded (ETX-I to ETX-6) in Table 7, only ETX-6 (86.33% ± 0.76) had less than 90% of the active ingredient. In this situation, a drug’s efficacy could be affected by an insufficient amount of the active ingredient. According to Indian pharmacopoeia, the percentage content of etoricoxib should fall within the range of 90%-110%.

**Table 7:** Results of analysis of 6 brands of etoricoxib by the proposed method.

Code	Tablet name	Labelled amount	% Recovery	% of added
ETX-I	Etricib tablet	90 mg	93.84 ± 0.70	99.87 ± 0.33
ETX-2	Etropain tablet	90 mg	93.11 ± 0.75	100.40 ± 0.54
ETX-3	Entorik tablet	90 mg	98.44 ± 0.29	100.50 ± 0.96
ETX-4	Etoheal tablet	90 mg	96.73 ± 0.08	100.09 ± 0.76
ETX-5	Arcorar tablet	90 mg	98.17 ± 1.08	100.22 ± 0.40
ETX-6	E-COX tablet	90 mg	86.33 ± 0.76	100.33 ± 0.35

### System suitability tests

To determine the system suitability of the proposed method, several parameters were examined, with the results shown in Table 8. These parameters included column efficiency (theoretical plates), capacity factor, resolution, tailing factor, selectivity, and percentage relative standard deviation of retention times.

**Table 8:**
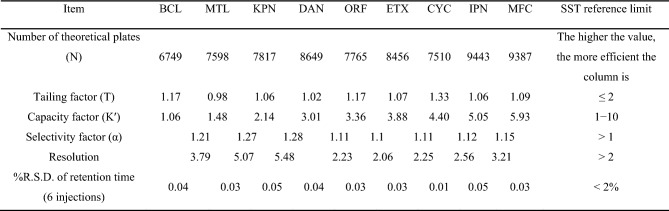
Results of system suitability tests for the optimized HPLC method.

### Validation of the optimized method

The principles of the ICH recommendations have been applied to the validation of the proposed method^65^.

#### Linearity

Eight different concentrations of the nine studied drugs were analyzed in triplicates to achieve linearity. The good linearities were confirmed by high values for the regression coefficients. Nine calibration curves with nine regression equations were constructed by plotting the relationship between drug concentrations and the corresponding peak areas. All data from calibration curves, such as values of slopes, intercepts, and regression coefficients, as well as standard deviations for the intercept and slope, are shown in Table 6.

#### Accuracy

To validate the accuracy of the proposed method, five different concentrations of the nine studied drugs were injected three times. Following this, the percent recoveries of the studied drugs were calculated in their pure standard mixtures. The content recoveries of the studied drugs in formulated dosage forms were calculated by the standard addition technique. As seen in Table 6, good recovery values were obtained for drugs in both pure standard mixtures and in formulated dosage forms.

#### Precision

The intraday precision of the proposed method was estimated by analysis of three concentrations of BCL, MTL, KPN, DAN, ORF, ETX, CYC, IPN, and MFC at 80, 100, and 120% as (20, 300, 80, 20, 24, 72, 12, 160, and 100 µg/mL), (25, 375, 100, 25, 30, 90, 15, 200, and 125 µg/mL), and (30, 450, 120, 30, 36, 108, 18, 240, and 150 µg/mL), respectively, three times on the same day. The inter-day precision (intermediate precision) was calculated using the same concentrations that were injected three times on three consecutive days. The R.S.D.s were determined to be less than 2%, as displayed in Table 6, demonstrating acceptable repeatability and intermediate precision of the proposed method.

#### Detection and quantitation limits

Using the method based on the response standard deviation (Sa) and slope of the calibration curve (b), the detection and quantitation limits were calculated by using the following equations: LOD = 3.3 Sa/b and LOQ = 10 Sa/b. The good results reflect the good sensitivity of the proposed method as shown in Table 6.

#### Selectivity

The term "selectivity" refers to a method's capacity to detect and measure an analyte's response without interference from other analytes and excipients. It was evaluated by the examination of several laboratory-made mixtures of the nine studied drugs, as well as through the analysis of various dosage forms of the studied drugs. It is found that the drug peaks in the sample solutions of all dosage forms are similar to those in the standard solutions. Additionally, no chromatographic impurity interference was seen in any of the dosage forms. The good resolution between the drug peaks and the successful determination of the nine studied drugs in pharmaceutical dosage forms, as shown in Fig. 6 and Table 6, both confirmed the good selectivity.

## Statistical analysis

The results obtained from the proposed method have been statistically analyzed using the Student's t-test and variance ratio F-test, at P = 0.05, with those obtained using the U.S.P. reference methods for BCL, MTL, KPN, DAN, ORF, CYC, IPN, and MFC^7^ and the I.P reference method for ETX^66^. The results show no significant difference in accuracy and precision between the methods for each drug, as shown in Table 9.

**Table 9:** Statistical analysis of the results obtained by the proposed method and the reference methods (V: variance; t(2.3): the critical t-value; F(6.9): the critical F-value at P = 0.05; Ref: reference method; Pro: proposed method).

Term	BCL		MTL		KPT		DAN		ORF		ETX		CYC		IPN		MFC	
	Ref	Pro	Ref	Pro	Ref	Pro	Ref	Pro	Ref	Pro	Ref	Pro	Ref	Pro	Ref	Pro	Ref	Pro
Mean%	100.63	100.21	100.35	100.44	100.44	100.07	100.08	99.94	100.45	100.23	99.89	99.70	100.67	100.34	100.08	100.53	100.22	100.30
S.D. ±	0.73	0.63	0.40	0.90	0.41	0.94	0.59	0.53	0.93	0.82	0.80	0.72	0.82	0.86	0.59	0.65	0.58	0.84
S.E. ±	0.33	0.28	0.18	0.40	0.17	0.42	0.26	0.24	0.42	0.37	0.36	0.32	0.37	0.38	0.26	0.29	0.26	0.38
R.S.D%	0.73	0.63	0.40	0.90	0.41	0.94	0.59	0.53	0.93	0.82	0.80	0.73	0.81	0.86	0.59	0.65	0.58	0.84
n	5	5	5	5	5	5	5	5	5	5	5	5	5	5	5	5	5	5
V	0.54	0.40	0.16	0.82	0.60	0.89	0.35	0.29	0.87	0.67	0.63	0.52	0.67	0.74	0.35	0.42	0.34	0.71
t(2.3)		0.96		0.70		0.97		0.39		0.56		0.40		0.63		1.15		0.17
F(6.9)		0.75		5.04		1.47		0.82		0.78		0.83		1.10		1.21		2.07

## Conclusion

In this work, screening, optimization, and robustness assessment were successfully carried out using experimental design (Plackett–Burman and Box-Behnken designs) to develop the RP HPLC method for the separation and simultaneous quantification of five drugs of skeletal muscle relaxants with four analgesic drugs (BCL, MTL, KPN, DAN, ORF, ETX, CYC, IPN, and MFC) in their pure standard mixtures and in pharmaceutical products. The suggested method was quick, precise, accurate, and sensitive, and it allowed the separation of the nine drugs in less than 8 min of satisfactory runtime. The suggested method was successfully used to estimate the concentrations of the analyzed drugs in pharmaceutical products after being fully validated by the validation process described in the ICH recommendations. Moreover, the proposed method revealed a lower quantity of the active ingredient ETX in Indian pharmaceutical products than declared on the label. It also confirmed the absence of counterfeit drugs in the analyzed massage cream. The suggested method can therefore be applied, particularly in laboratories of quality control, for routine evaluation of pharmaceutical products containing the cited drugs and can also be applied to detect the potential counterfeiting of these drugs.

### Supplementary Information

Supplementary Information.

## Data Availability

All data generated or analyzed during this study are included in this published article.

## Abbreviations

AQbD: Analytical quality by design
ATP: Analytical target profile
ADS: Analytical design space
ACN: Acetonitrile
A: Acetonitrile%
B: Flow rate
BBD: Box-Behnken design
BCL: Baclofen
CAAs: Critical analytical attributes
CMVs: Critical method variables
COX-2: Cyclooxygenase-2
CYC: Cyclobenzaprine HcL
QRM: Quality risk management
ICH: International Council for Harmonisation
C: Buffer pH
DoE: Design of experiment
D: Acetate buffer concentration
DAN: Dantrolene sodium
ETX: Etoricoxib
GABA-B: γ-Aminobutyric acid
GC–MS: Gas chromatography-mass spectrometry
HPLC: High-performance liquid chromatography
HPTLC: High-performance thin-layer chromatography
IPN: Ibuprofen
I.P: Indian pharmacopeia
IR: Infra-red
KPN: Ketoprofen
LC–MS: Liquid chromatography-mass spectrometry
LOD: Limit of detection
LOQ: Limit of quantitation
MFC: Mefenamic acid
MTL: Methocarbamol
NSAID: Non-steroidal anti-inflammatory drug
NMR: Nuclear magnetic resonance
ORF: Orphenadrine citrate
p-value: Probability value at 95%
RP-HPLC: Reversed-phase high-performance liquid chromatography
PBD: Plackett–Burman design
TLC: Thin-layer chromatography
WHO: World Health Organization
U.S.P.: United States Pharmacopeia
UV: Ultraviolet
tR-9: The retention time of mefenamic acid
mM: Milimole
R-1: Resolution between BCL and MTL peaks
R-2: Resolution between MTL and KPN peaks
R-3: Resolution between KPN and DAN peaks
R-4: Resolution between DAN and ORF peaks
R-5: Resolution between ORF and ETX peaks
R-6: Resolution between ETX and CYC peaks
R-7: Resolution between CYC and IPN peaks
R-8: Resolution between IPN and MFC peaks
