# Supplementary material for: Utilizing experimental design and desirability function in optimizing RP-HPLC method for simultaneous determination of some skeletal muscle relaxants and analgesics
Source: Sci Rep. 2024 May 6;14:10360. doi: 10.1038/s41598-024-58381-4 (PMC11074291; doi:10.1038/s41598-024-58381-4)
Supplement: Supplementary file 1 — Supplementary Information. [file 41598_2024_58381_MOESM1_ESM.docx]

**Supplementary Tables**

**Supplementary Table S1** The different pharmaceutical products. ^a^: represents pharmaceutical products purchased from Yemeni markets.

| **Trade name** | **Active constituents and strength** | **Producers** |
| --- | --- | --- |
| MYLOBAC 25 mg tablets | Baclofen 25 mg | PHARAONIA PHARMACEUTICALS Company (Cairo, Egypt) |
| METHOCARBEX 750/400 mg tablets | Methocarbamol 750 mg + Ibuprofen 400 mg | EGYPHAR Company (Cairo, Egypt) |
| KETOLGIN 200 mg capsules | Ketoprofen 200 mg | AMOUN PHARMACEUTICAL Company (Cairo, Egypt) |
| DANTRELAX 25 mg capsules | Dantrolene sodium 25 mg | CHEMIPHARM Company (Cairo, Egypt) |
| NORFLEX 30 mg/1mL ampoule | Orphenadrine citrate  (each mL contains 30 mg) | EIPICO Company (Tenth of Ramadan City, Egypt) |
| ARCOXIA 90 mg tablets | Etoricoxib 90 mg | MSD (Merck Sharp & Dohme) Company (Cairo, Egypt) |
| MULTI-RELAX 10 mg tablets | Cyclobenzaprine HCl 10 mg | APEX PHARMA (Cairo, Egypt) |
| MEFENTAN 250 mg capsules | Mefenamic acid 250 mg | GLOBAL NAPI (Cairo, Egypt) |
| ETRICIB-90 tablets ^a^ | Etoricoxib 90 mg | ZIM Laboratories Limited (Maharashtra state, India) |
| ETROPAIN-90 tablets ^a^ | Etoricoxib mg | SIGNATURE PHYTOCHEMICAL Industries (Dehradum, India) |
| ENTORIK-90 tablets ^a^ | Etoricoxib 90 mg | RIVPRA Formulation Pvt.Ltd (Uttarakhand, India), |
| ETOHEAL-90 tablets ^a^ | Etoricoxib 90 mg | MASCOT Health Series Pvt.Ltd (Haridwar, India), |
| ARCORAR-90 tablets ^a^ | Etoricoxib 90 mg | AMBIX Healthcare LLP (Gujarat, India), |
| E-COX-90 tablets ^a^ | Etoricoxib 90 mg | VAPI CARE PHARMA Pvt.Ltd (Gujarat, India) |
| MYOCOOL massage cream ^a^ | Salicylic acid, mineral oil, camphor oil, stearic acid, eucalyptus oil, menthol, and peppermint oil | LEADER for Cosmetics and Perfumes (Giza, Egypt) |
| ROTMOOV massage cream | Menthol crystals, camphor oil, clove oil, paraffin oil, and stearic acid | Hi Care Pharmaceutical and Cosmetics (Cairo, Egypt) |
| MOOV massage topical cream | Oleoresin capsicum, camphor, menthol, methylsalicylate, and camphor oil | EVA PHARMA (Giza, Egypt) |
| ALGASON massage cream | Diethylamine salicylate, camphor, and menthol | PHARCO PHARMACEUTICALS (Alexandria, Egypt), |
| MESTAVIL massage gel | Camphor, and menthol, eucalypus oil, carbomeer, and triethanol amine | NIKIM SPECIAL CHEMICALS (Giza, Egypt) |

| **Supplementary Table S2** Analysis of Variance (ANOVA) of PBD. *: represents the non-significant values. | | | | | | | |
| --- | --- | --- | --- | --- | --- | --- | --- |
| **Response** | **Statistical parameter** | **Model** | **Linear** | **A** | **B** | **C** | **D** |
| t_R_-9 | F-Value | 100.87 | 100.87 | 158.29 | 239.51 | 5.67 | 0.000 |
|  | P-Value | 0.000 | 0.000 | 0.000 | 0.000 | 0.049 | 0.958* |
| R-1 | F-Value | 227.45 | 227.45 | 769.55 | 139.33 | 0.52 | 0.41 |
|  | P-Value | 0.000 | 0.000 | 0.000 | 0.000 | 0.492* | 0.543* |
| R-2 | F-Value | 165.15 | 165.15 | 317.25 | 83.42 | 14.94 | 244.97 |
|  | P-Value | 0.000 | 0.000 | 0.000 | 0.000 | 0.006 | 0.000 |
| R-3 | F-Value | 133.79 | 133.79 | 255.79 | 71.29 | 11.46 | 196.62 |
|  | P-Value | 0.000 | 0.000 | 0.000 | 0.000 | 0.012 | 0.000 |
| R-4 | F-Value | 29.67 | 29.67 | 4.56 | 10.87 | 101.65 | 1.58 |
|  | P-Value | 0.000 | 0.000 | 0.07 | 0.013 | 0.000 | 0.249* |
| R-5 | F-Value | 34.51 | 34.51 | 25.41 | 1.82 | 109.45 | 1.36 |
|  | P-Value | 0.000 | 0.000 | 0.001 | 0.219* | 0.000 | 0.282* |
| R-6 | F-Value | 32.99 | 32.99 | 0.16 | 12.56 | 105.14 | 14.1 |
|  | P-Value | 0.007 | 0.007 | 0.70* | 0.009 | 0.000 | 0.007 |
| R-7 | F-Value | 22.52 | 22.52 | 19.07 | 2.76* | 66.34 | 1.91 |
|  | P-Value | 0.000 | 0.000 | 0.003 | 0.14 | 0.000 | 0.210* |
| R-8 | F-Value | 100.25 | 100.25 | 307.47 | 29.63 | 0.62 | 63.3 |
|  | P-Value | 0.000 | 0.000 | 0.000 | 0.001 | 0.458* | 0.000 |

| **Supplementary Table S3** Predictive and experimental values of different responses under optimal conditions | | | | | | | |
| --- | --- | --- | --- | --- | --- | --- | --- |
| Response | Prediction | Experimental | SE mean | 95% CI | | 95% PI | |
|  |  |  |  | low | high | low | high |
| t_R-6_ | 6.648 | 6.702 | 0.065 | 6.512 | 6.785 | 6.416 | 6.881 |
| R-1 | 3.801 | 3.793 | 0.037 | 3.724 | 3.877 | 3.628 | 3.973 |
| R-2 | 5.428 | 5.068 | 0.050 | 5.325 | 5.532 | 5.199 | 5.658 |
| R-3 | 5.167 | 5.483 | 0.060 | 5.043 | 5.292 | 4.904 | 5.431 |
| R-4 | 2.264 | 2.230 | 0.086 | 2.086 | 2.443 | 1.823 | 2.705 |
| R-5 | 2.147 | 2.063 | 0.056 | 2.031 | 2.263 | 1.885 | 2.409 |
| R-6 | 2.146 | 2.252 | 0.070 | 2.002 | 2.290 | 1.715 | 2.409 |
| R-7 | 2.996 | 2.563 | 0.159 | 2.666 | 3.327 | 2.250 | 3.743 |
| R-8 | 3.111 | 3.208 | 0.032 | 3.044 | 3.178 | 2.983 | 3.238 |

**Supplementary Figure**


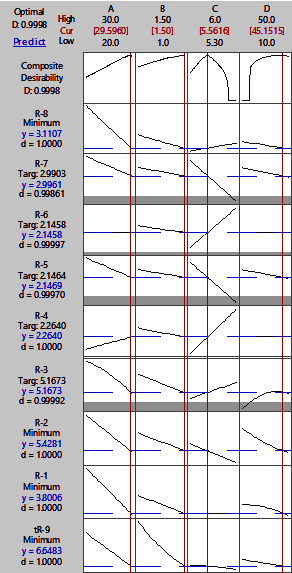


**Supplementary Fig. S1.** The optimization plot for the measured responses generated by Minitab.
